# The Accuracy, Feasibility and Challenges of Sequencing Short Tandem Repeats Using Next-Generation Sequencing Platforms

**DOI:** 10.1371/journal.pone.0113862

**Published:** 2014-12-01

**Authors:** Monika Zavodna, Andrew Bagshaw, Rudiger Brauning, Neil J. Gemmell

**Affiliations:** 1 Department of Anatomy, University of Otago, Dunedin, New Zealand; 2 Department of Pathology, University of Otago, Christchurch, New Zealand; 3 AgResearch Limited, Invermay Agricultural Centre, Mosgiel, New Zealand; 4 Allan Wilson Centre for Molecular Ecology and Evolution, University of Otago, Dunedin, New Zealand; University of Torino, Italy

## Abstract

To date we have little knowledge of how accurate next-generation sequencing (NGS) technologies are in sequencing repetitive sequences beyond known limitations to accurately sequence homopolymers. Only a handful of previous reports have evaluated the potential of NGS for sequencing short tandem repeats (microsatellites) and no empirical study has compared and evaluated the performance of more than one NGS platform with the same dataset. Here we examined yeast microsatellite variants from both long-read (454-sequencing) and short-read (Illumina) NGS platforms and compared these to data derived through Sanger sequencing. In addition, we investigated any locus-specific biases and differences that might have resulted from variability in microsatellite repeat number, repeat motif or type of mutation. Out of 112 insertion/deletion variants identified among 45 microsatellite amplicons in our study, we found 87.5% agreement between the 454-platform and Sanger sequencing in frequency of variant detection after Benjamini-Hochberg correction for multiple tests. For a subset of 21 microsatellite amplicons derived from Illumina sequencing, the results of short-read platform were highly consistent with the other two platforms, with 100% agreement with 454-sequencing and 93.6% agreement with the Sanger method after Benjamini-Hochberg correction. We found that the microsatellite attributes copy number, repeat motif and type of mutation did not have a significant effect on differences seen between the sequencing platforms. We show that both long-read and short-read NGS platforms can be used to sequence short tandem repeats accurately, which makes it feasible to consider the use of these platforms in high-throughput genotyping. It appears the major requirement for achieving both high accuracy and rare variant detection in microsatellite genotyping is sufficient read depth coverage. This might be a challenge because each platform generates a consistent pattern of non-uniform sequence coverage, which, as our study suggests, may affect some types of tandem repeats more than others.

## Introduction

Recent technical developments and improvements in next-generation sequencing (NGS) platforms have revolutionized genetic and genomic research by increasing the throughput and lowering the cost of DNA sequencing. NGS technology makes it possible to sequence millions of individual DNA strands in DNA mixtures such as fragmented genomic DNA or multiplexed amplicons in relatively short time [1], [2]. This technology has found application in basic, applied and clinical research, spanning whole genome sequencing of model and non-model organisms, transcriptomics and differential gene expression (RNA-seq), chromatin immunoprecipitation followed by sequencing (ChIP-seq), and as a tool to identify and validate new genetic markers and mutations [3]–[5]. Current NGS platforms produce shorter reads than Sanger sequencing, but with much greater read numbers, which increases complexity. Consequently, the development of these new sequencing methods has been tightly coupled with developments in computational tools and bioinformatics [6].

One of the biggest technical challenges that are associated with NGS technologies are repetitive DNA sequences [7]. Treangen and Salzberg [6] reviewed computational challenges posed by repeat sequences for genome re-sequencing projects, *de novo* genome assembly and RNA-seq analysis. They focused on repeat classes with sequence lengths of at least 100 bp, that occur two or more times in the genome and exhibit>97% identity to at least one other copy of themselves, and they concluded that such repeats result in ambiguities in alignment and assembly that may lead to biases and errors in interpretation. While not the focus of that work, the difficulties posed by repeats and the computational errors they generate would surely be exacerbated by any intrinsic errors in the sequencing of these repeats. However, to date we have little knowledge of how accurate NGS approaches are in sequencing repetitive sequences beyond known limitations accurately sequencing homopolymer runs [8], [9].

Microsatellites are short (typically less than 100 bp) DNA sequences in which motifs of 1–6 bp are tandemly repeated. They are highly polymorphic in their length, which results from variability in repeat number [10], [11]. This has led them to be broadly used as genetic markers in various research areas, particularly in population and conservation genetics, molecular ecology, forensics, parentage analyses and gene mapping [12]. While most microsatellites are thought to be selectively neutral, mutations in the lengths of some functional microsatellites are responsible for, or strongly associated with more than 20 human neurological disorders and diseases [13]–[15].

The analysis of microsatellite polymorphism has traditionally been based on locus specific PCR-amplification followed by fluorescence capillary electrophoresis to identify fragment length differences [16]. High-throughput NGS technology offers a number of potential advantages for microsatellite genotyping. First, it provides sequence data, which allows discrimination of the alleles with the same length and base pair composition, but with different repeat structures, potentially circumventing issues of homoplasy that have affected interpretations of microsatellites in the past [17]. Second, it also allows detection of substitutions and single nucleotide polymorphisms (SNPs) in the repeats or adjacent DNA regions. Furthermore, the microsatellite sequences generated by NGS can be identified based on the flanking or primer sequences and therefore, the number of loci in a single analysis is not limited by number of fluorescent dyes and/or non-overlapping size ranges of the loci.

Despite rapid and ubiquitous implementation of NGS platforms in genetics and genomics, only a handful of previous reports have evaluated the accuracy of these platforms for sequencing tandem repeats and their potential for microsatellite genotyping [15], [18]–[22]. Most of these were human forensic studies, using the GS FLX 454-sequencing platform, which generates longer reads (400–500 bp, currently up to 1000 bp) and is therefore considered better suited for obtaining full length reads of microsatellite amplicons than other NGS platforms [18], [20], [21]. These authors demonstrated the feasibility and reliability of NGS for microsatellite genotyping when compared to traditional capillary electrophoresis [18], [20] and highlighted the advantage of the NGS technology in detecting nucleotide substitutions and repeat structure variations that normally go undetected using capillary electrophoresis.

Due to the short length of sequencing reads obtainable from the Illumina platform (150–250 bp), this NGS technology had been assumed impractical for microsatellite genotyping. However, Bornman et al. [19] since demonstrated that highly accurate microsatellite genotyping is also possible on this platform. A recent review by Cao et al. [22] compared and evaluated the utility of several NGS technologies, including long-read and short-read platforms for accurately detecting short tandem repeat variation from whole genome sequencing data. The authors suggested that long read-length and/or paired-end sequencing with a tight fragment length distribution be used to optimise the probability of detecting microsatellite variants with high accuracy in genomic data. However, their evaluation was based on simulated data and validated on only one NGS sequencing platform. The validation was done through testing for the presence of predicted deletion/insertion polymorphisms using agarose gel electrophoresis, but the accuracy in determining the lengths of the insertions and deletions was not tested [23]. Thus, to date, no empirical study has compared and evaluated the performance of more than one NGS platform for detecting short tandem repeat variation in the same dataset.

Here we undertook the first comprehensive empirical study that examines and compares the performance of multiple NGS platforms for short tandem repeat sequencing. Using a reference set of yeast DNA samples we evaluate both long-read (454-sequencing) and short-read (Illumina) NGS platforms and compare these to data derived through Sanger sequencing. Unlike previous studies, our experimental design allowed us to (1) examine microsatellite variants from both amplicon and whole genome next-generation sequencing and (2) investigate any locus-specific biases and differences that might have resulted from variability in microsatellite copy number, repeat motif or type of mutation.

## Materials and Methods

### Study species and DNA extraction

We used 16 populations of *Saccharomyces cerevisiae* derived from the haploid DH89 *α, ho* strain, which in turn derives from the laboratory Y55 wildtype strain [24]. For each population, genomic DNA was extracted from yeast culture regrown in a liquid YPD medium overnight. Briefly, cultured yeast cells were pelleted, resuspended in a spheroplastic solution (1 M sorbitol, 20 mM EDTA, 10 mM Tris-HCl pH 7.4) containing 40 U/ml of lyticase and 1 µl/ml of ß-mercaptoethanol and incubated at 37°C shaking for one hour. After incubation, the yeast lysate was again pelleted, resuspended in a buffer containing 50 mM Tris-HCl pH 7.4, 20 mM EDTA and 1% SDS and further incubated at 65°C for 30 min. To precipitate proteins, 5 M potassium acetate (1/5 of the lysate volume) was added to the lysate, which was then vortexed and placed on ice for one hour. The lysate was then centrifuged and supernatant containing DNA was precipitated with isopropanol. The DNA was recovered by high-speed centrifugation. After the DNA pellet was washed with 70% ethanol twice, it was air-dried and dissolved in TE buffer (10 mM Tris-HCl, pH 8.0, 0.1 mM EDTA). The genomic DNA samples were further purified using 5 M lithium chloride and chloroform as described in Gemmell and Akiyama [25].

### Identification and selection of microsatellite loci

We identified microsatellites with 2–6 bp repeat motifs in the *S. cerevisiae* genome (strain S288c, GenBank NC_001133 through NC_001148) using a program written in C as described in Bagshaw et al. [26]. For motif sizes of 2–3 bp we used a minimum repeat number of six, while for motif sizes of 4–6 bp we lowered the threshold to four repeats due to their low frequency in the genome. One mismatch to a consensus motif was allowed for every ten bp of uninterrupted microsatellite sequence. In total we detected 255 microsatellite loci that were also confirmed in yeast strain Y55, the genome sequence of which became available during the course of this study (ftp://ftp.sanger.ac.uk/pub/users/dmc/yeast/latest/). Locus-specific PCR primers, designed for all microsatellite loci using Primer 3 [27], were used unmodified in the amplifications for conventional Sanger sequencing.

To facilitate GS FLX 454-sequencing (Roche Diagnostics), the primers were modified into fusion primers, which consisted of: (1) locus-specific microsatellite primers; (2) multiplex identifier sequence (MID, Roche Diagnostics); and (3) the Roche GS FLX 454-sequencing adaptors [28]. The use of MID sequences (tags/barcodes) enabled parallel high-throughput 454 sequencing of multiple samples and their subsequent identification.

### PCR amplifications for Sanger sequencing and 454-sequencing

For each sample, each microsatellite locus was amplified by individual polymerase chain reaction (PCR). All amplification reactions were performed in 25 µl volume containing 10 ng of template genomic DNA, 1x NH_4_ reaction buffer (16 mM (NH_4_)_2_SO_4_, 67 mM Tris-HCl pH 8.8, 0.01% stabilizer; supplied with BIOTAQ DNA Polymerase), 3 mM MgCl_2_, 1 µM of each primer (primer type depended on the type of sequencing technique; for details see above), 200 µM of each dNTP and 1 Unit BIOTAQ DNA Polymerase (Bioline). PCR conditions were 94°C for 5 min followed by 32 cycles of 94°C for 30 s, 60°C for 30 s, 72°C for 30 s, and a final extension at 72°C for 10 min. The PCR products were verified on a 1% agarose gel stained with SYBR Safe.

All successfully amplified PCR products were purified using Agencourt AMPure XP DNA purification kit following the manufacturer's instructions (Beckman Coulter). DNA concentrations of the purified amplicons were measured using Quant-iT PicoGreen dsDNA Assay kit (Invitrogen, Molecular Probes).

### Next-generation sequencing (Illumina and 454 sequencing) and Sanger sequencing

From our sample of 16 yeast populations, 12 of these were also sequenced through the Illumina FastTrack Service (Hayward, USA). Whole genome sequencing using 100 bp paired-end reads of libraries with an average insert size 300 bp was undertaken on one lane of an Illumina Genome Analyzer IIx using chemistry version 5. This design expected around 40-fold average coverage depth per genome.

For GS FLX 454 sequencing, equimolar amounts of all purified barcoded amplicons (i.e. amplified using the fusion primers) were pooled and submitted for 454-sequencing at the High Throughput Sequencing Service (University of Otago, New Zealand).

A subset of 50 purified PCR products that had been amplified using the locus-specific primers were submitted for cloning and Sanger sequencing at the Central Analytical Facility (Stellenbosch University, South Africa) and Macrogen Inc. (Seoul, South Korea). Ten clones per amplicon were Sanger sequenced in both forward and reverse directions, which provided 20 unambiguous sequences per PCR product.

### Data analyses - Illumina sequencing

For each of the 12 samples, the quality of obtained reads was first examined using FastQC software (Babraham Institute) with default settings. Low quality bases were trimmed off using Dynamic Trim software with default settings [29]. Subsequently, trimmed reads were aligned to the Y55 yeast genome reference (ftp://ftp.sanger.ac.uk/pub/users/dmc/yeast/latest/) using Burrows-Wheeler Alignment Tool version 0.5.9-r16 with default settings [30]. The SAM format alignments obtained for each sample were converted into sorted BAM files using SAM tools [31], which were then further processed using CLC Genomics Workbench 6.0.5 (*CLC bio*). Aligned reads were mapped to the 255 microsatellite reference loci. The CLC mapping parameters were set to the following: mismatch, insertion and deletion cost  = 2, length fraction  = 0.75, similarity fraction  = 0.9 and non-specific matches were set to ignored. This filtering included in the final mappings only read alignments that had greater than 90% similarity with the reference in at least 75% of their read length and non-specific matches, i.e. the reads that would align at more than one position with an equally good score, were excluded from the final mappings. It is important to note that the CLC Genomics Workbench mapping parameters do not filter reads based on the read length per se, thus reads of various lengths may be included in the final mappings that are then used for variant detection. Nevertheless, insertion and deletion variants are only detected if they are spanned by reads, which means that the reads have to align both before and after the variant (*CLC bio*). The quality filters for the quality-based variant detection were set to neighborhood radius  = 5, maximum gap and mismatch count  = 2 and minimum central and neighboring Phred quality score  = 20. The significance thresholds were set to minimum coverage  = 5 reads and a minimum variant frequency  = 10%. Thus, low frequency variants/alleles that may result from sequencing errors [32] or represent microsatellite stutters [20] were excluded from our analyses. The detected variants were filtered and only InDel (Insertion and Deletion polymorphism) variants with length of 2 bp and greater were further considered.

### Data analyses – 454-sequencing

The reads obtained from GS FLX 454-sequencing were initially examined using the Genome Sequencer FLX System Software version 2.3 and its SFF Tools commands (Roche Diagnostics). Only reads that contained complete MID barcodes with no mismatches were extracted for the analysis. The extracted Standard Flowgram Format (sff) files were then processed using CLC Genomics Workbench 6.0.5 (*CLC bio*). Reads per each sample were mapped against the 255 microsatellite reference loci. The CLC mapping parameters and the criteria for the quality-based variant detection were almost identical to those used for the Illumina sequencing reads described above, but the minimum coverage was set to 25 reads and the minimum variant frequency set to 5%. These adjustments accounted for the higher average coverage per locus obtained from the amplicon 454-sequencing compared to the whole genome Illumina sequencing and aimed to achieve a similar level of variant detection across the platforms. Only InDel variants with length of 2 bp and greater were further considered. In addition, the variants in homopolymers were excluded from further analyses, as 454-sequencing is known to have difficulties in accurately sequencing these regions [8], [9].

### Data analyses - Sanger sequencing

To eliminate alignment software bias or errors, the microsatellite sequences obtained from Sanger sequencing were also analyzed using the CLC Genomics Workbench 6.0.5 (*CLC bio*). The analyses were performed as described with the following modifications: using the functions specific to Sanger sequence data, the sequences for each amplicon were assembled against the relevant microsatellite reference locus using default parameters. The criteria for the quality-based variant detection were the same as those used for the Illumina sequencing reads described above, apart from minimum coverage, which was set to 10 reads and a minimum variant frequency set to 5%. These modifications were again derived from the average coverage per locus obtained from our Sanger sequencing and aimed to achieve a similar level of variant detection across the platforms. Only InDel variants with length of 2 bp and greater were further considered in our analyses.

### Comparison of microsatellite polymorphism detection using three sequencing platforms

Although the NGS sequencing on both platforms resulted in a large number of microsatellite loci sequenced in total, in this analysis we focus only on the subset of amplicons (n = 45) that were also successfully sequenced using the Sanger method. These amplicons represented 21 microsatellite loci, with an average amplicon length 138.8±24.7 bp (mean ± SD). In order to assess how feasible and accurate NGS platforms are for high-throughput parallel microsatellite-amplicon sequencing we compared InDel variant data obtained from the NGS platforms to those obtained from the conventional Sanger sequencing. Statistical tests were performed using functions available in R statistical software version 3.0.3 [33]. Probabilities that different sequencing platforms detected variants with the same frequency were calculated using the chi-square or log-likelihood goodness of fit tests. For each variant and platform, chi-square testing was done in the standard way for a 2×2 contingency table (2×3 for the three-way comparisons), in which columns corresponded to two (or three) platforms and rows corresponded to counts of variant and non-variant reads. To obtain expected read counts, the row and column totals were multiplied and the result was divided by total number of reads across the platforms. For variants where one or more expected read counts were less than one, we used chi-square with Monte-Carlo simulation in R (10000 re-samplings per test) to calculate P values [34]. We opted for this, because the standard chi-square test is known to be less accurate in these cases [35], and while Fisher's Exact Test (Fisher-Irwin test) is often recommended as a substitute, our data does not meet its requirement of fixed marginal totals. In cases where the observed number of reads minus the expected number was ≥ the expected number and other criteria for the use of the standard chi-square test were met, we used a log-likelihood ratio goodness of fit test as described by Zar [35]. We did not perform combined tests on the sums of observed and expected read counts for all variants found by each platform because heterogeneity chi-square analysis showed very clear heterogeneity in all cases (all p<0.0001). For false discovery correction we used both the conservative Bonferroni and less conservative Benjamini-Hochberg methods [36].

## Results

The two NGS strategies, amplicon (454-sequencing) and whole genome (Illumina) sequencing differed in the number of microsatellite loci that passed our quality criteria for InDel variant detection analysis. This was largely caused by differences in coverage, as the average coverage for loci sequenced by 454-platform and analyzed in this study was 77.2 reads, while the average coverage for loci sequenced by Illumina in this study was 13.8 reads. As a result of this, less than half of the variants detected by both Sanger and 454-sequencing were detected by Illumina platform.

Therefore, our first point of comparison in validating microsatellite mutation detection by NGS platforms was between 454-sequencing and Sanger sequencing. Among the 45 amplicons that represented 21 yeast microsatellite loci, we detected a total of 112 InDel variants using the two sequencing methods (Table S1). There was no significant difference between these methods in observed variant frequencies overall (p = 0.53, Wilcoxon rank test; Table S1). However, of the 112 variants detected, 37 were identified by 454-sequencing but not Sanger and 18 were identified by Sanger but not 454-sequencing (Table S1). It was apparent that stochastic effects caused by the higher coverage obtained on the 454-platform and/or the rareness of some variants could account for some of these differences, so we used goodness of fit testing in order to identify which variants showed statistically significant disagreement between platforms. Significant differences (all p<0.05) were found for 29 of the 112 variants (25.9%), which was clearly greater than the number expected by chance (5.6 at α = 0.05). Nine variants (8%) remained significantly different after Bonferroni correction, and 14 (12.5%) remained significant after Benjamini-Hochberg correction (Table S1).

We looked further for locus-specific biases by comparing attributes/contexts of the variants for which significant differences were found between platforms with those for which no difference was found. Initially, we compared the 29 variants showing nominally significant detection frequency differences between the two platforms with the remaining 83 variants. We found no significant differences with respect to InDel type defined as number of repeats deleted or inserted (χ^2^ = 4.06, p = 0.54), InDel type defined as deletion or insertion (χ^2^ = 0.19, p = 0.66), microsatellite repeat number (p = 0.50; Wilcoxon rank test) or microsatellite motif (χ^2^ = 0.74, p = 0.69). We also found no significant results repeating these tests and comparing the subset of variants, for which detection differed among platforms after Bonferroni and Benjamini-Hochberg corrections, with remaining variants.

Of the 29 InDel variants detected differently by Sanger and 454-sequencing at α = 0.05, eleven were not detected by 454-sequencing but only four of these eleven were present in more than four Sanger sequences or two clones. These four mutation sites had relatively poor coverage by 454-sequencing, with an average of 34.3 reads, well below the overall 454-platform average of 77.2 reads. However, the 29 variants that differed in detectability across platforms, when considered together, did not have significantly different coverage from the remaining variants in 454-sequencing (p = 0.36; Wilcoxon rank test).

Sanger coverage was relatively uniform across all variants (18±3.2), but 454-sequencing coverage varied substantially (77.2±55). Searching for modifiers of 454-platform coverage over all detected variants, we found that motif type was not a significant predictor of coverage (p = 0.059; Kruskal-Wallis test). However, we did find that coverage of AC microsatellite variants (n = 17) was significantly lower, with an average of 47.3 reads, compared to AT microsatellites variants (n = 87) showing an average of 82.1 reads (p = 0.014; Wilcoxon rank test). It was also apparent that the 454-platform may have been somewhat poorer at detecting AC microsatellite variants independently of coverage, as the observed frequencies of these variants were nearly twice as high on average using Sanger sequencing, but this was not significant (p = 0.057; Wilcoxon rank test; Table S1). Microsatellite repeat number had no significant effect on 454-platform coverage (p = 0.168; Spearman's rho).

**Figure 1:**
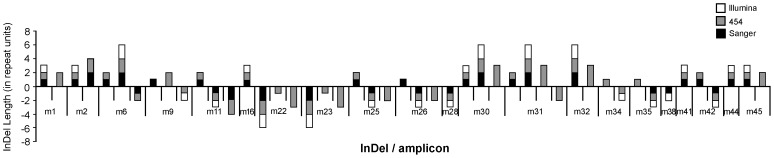
Summary of detected microsatellite InDel mutations from three sequencing platforms. Plot of the microsatellite InDel variants per amplicon (m) detected by Illumina- (white bars), 454-sequencing (grey bars) and Sanger sequencing (black bars). Insertions are denoted by bars above the X-axis and deletions are denoted by bars below the X-axis. The length of each bar segment is proportional to the length of the InDel in repeat units inserted or deleted. The amplicons' characteristics are summarized in Table S1.

Our second point of comparison in validating microsatellite variant detection by NGS platforms was with Illumina sequencing. Due to low average coverage from yeast whole genome sequencing, only a subset of amplicons (n = 21) was included in this analysis (Figure 1, Table S1). Thus, of the 112 variants detectable by 454-platform and Sanger sequencing, only 47 were also detectable by Illumina sequencing, with an average coverage of 13.8 reads. In three-way comparisons between Illumina, 454- and Sanger sequencing we found that 11 out of 47 variants differed significantly (p<0.05; Table S1). Seven differences remained significant after Benjamini-Hochberg correction and four remained significant after Bonferroni correction (Table S1). These variants were not significantly different from the remaining variants by motif, InDel type or repeat number (p>0.05). In two-way comparisons between Illumina and Sanger sequencing and between Illumina and 454-sequencing (methods as described above for Sanger vs 454-sequencing) six significant differences were found in both cases (p<0.05), but only three and none, respectively, remained significant after Benjamini-Hochberg correction. There was no clear evidence that the results produced by Illumina were closer to either Sanger or 454-sequencing results. Of the 11 differences found in the three-way comparisons, only one was seen in Sanger vs Illumina, but not 454-platform vs Illumina comparisons, and only two were seen in 454-sequencing vs Illumina but not Sanger vs Illumina comparisons.

Illumina sequencing gave more uniform coverage than 454-sequencing (13.8±5.2; Table S1). Unlike the 454-platform, there was no evidence for the Illumina platform having any difficulty with replicating poly-AC microsatellites, but there was slightly lower coverage of poly-AT repeats: the Illumina platform covered poly-AC with an average of 15.2 reads, which was greater than its average of 13.8 reads for poly-AT (p = 0.036 Wilcoxon rank test, though this was not significant by t-test, p = 0.15). All but one variant identified by Illumina sequencing were in AC or AT microsatellites.

## Discussion

In this study, we evaluated the application of two NGS platforms for accurately assessing microsatellite repeat sequence variation and genotyping in the same set of yeast DNA samples. We examined both long-read (454-sequencing) and short-read (Illumina) NGS platforms and compared these to Sanger sequencing. The 454-platform was used for high-throughput parallel sequencing of yeast microsatellite-specific amplicons, while the Illumina platform was used for yeast whole-genome sequencing from which we subsequently extracted mapped microsatellite loci of interest. Data from all sequencing techniques were analysed with the same mapping/InDel detection software and the parameters used were standardized in order to limit discrepancies that might arise from the data analysis [6].

Our experimental designs for Illumina sequencing expected around 40-fold average coverage depth per genome. From our obtained data, the mean raw coverage depth per genome was 38.1 reads, which decreased to a mean of 29.3 reads after trimming and alignment. As previously shown, however, each NGS technology generates a pattern of non-uniform sequence coverage [37]. For short-read NGS platforms in particular, the read depth coverage often decreases with increasing AT-rich repetitive sequences [37]. Indeed, the average coverage for microsatellite loci sequenced by Illumina in this study was 13.8 reads, while the mean coverage depth per genome overall was 29.3 reads. Hence, observed average coverage for the loci analyzed in this study was over 5-fold higher by the 454-platform compared to Illumina, which resulted in differences between the two NGS strategies in number of microsatellite loci that passed our quality criteria for InDel variant detection analysis. Thus, Illumina sequencing presented a challenge for our microsatellite analysis, but we believe this low coverage can be overcome by using this platform for direct microsatellite amplicon sequencing instead of whole genome sequencing with subsequent microsatellite reference mapping, or by more in-depth whole genome sequencing that would compensate for non-uniform coverage depth. Moreover, with ongoing rapid increases in read lengths being achieved across many NGS platforms including the Illumina platforms, non-uniform coverage may be improved even for whole genome sequencing in the near future. In Illumina sequencing for example, paired-end sequencing where read pairs overlap and can be fused into a single long read for each insert, could be beneficial for sequencing longer microsatellite loci. On the other hand, sequencing short tandem repeats would not profit from non-overlapping paired-end reads, because of difficulties with alignment to reference [19]. Thus, it is desirable that amplicon length, or insert size of the libraries in the case of whole genome sequencing, coincides with the read length of the instrument.

Out of 112 InDel variants identified among 45 microsatellite amplicons in our study, we found 87.5% agreement between the 454-platform and Sanger sequencing after Benjamini-Hochberg correction for multiple tests and 92% after Bonferroni correction. Out of 14 significant disagreements following Benjamini-Hochberg correction, six resulted from detection by Sanger sequencing but not by 454-sequencing. To further understand the cause of this discrepancy, we scrutinized the NGS mappings by eye. In all but one case we found that the variants were in fact present in the mappings, but had gone undetected due to their low frequency (<5%) in our 454-data. Similar discrepancies were previously reported and attributed to low coverage combined with allelic amplification bias [15]. Indeed, the variants in our study that had gone undetected in our 454-data were heterozygous, had greater numbers of microsatellite units and were sequenced at lower coverage depth than the average microsatellite amplicon.

Although our dataset for the Illumina microsatellite genotyping analysis was considerably smaller than that of the 454-platform, the short-read platform appeared to be highly accurate in InDel variant detection, with over 100% agreement with 454-sequencing and over 93% agreement with Sanger sequencing after Benjamini-Hochberg correction. Similar results were also demonstrated by the study of Bornman et al. [19].

In contrast to those few studies that have previously evaluated application of NGS for microsatellite genotyping [15], [18], [20], [21], our experimental study design also allowed us to investigate microsatellite-type-specific biases in sequencing accuracy using a long-read NGS platform. We found that the microsatellite attributes copy number, repeat motif and type of mutation did not cause the differences seen between the sequencing platforms. This suggested that depth of coverage might be the main consideration affecting the accuracy of microsatellite genotyping using NGS.

Interestingly, searching for modifiers of 454-platform coverage over all detected mutations, our study indicates that the 454-platform may have been somewhat poorer at sequencing AC microsatellite variants, as these had on average significantly lower coverage than AT microsatellite variants. On the other hand, microsatellite repeat number, the attribute that might have been expected to affect the NGS coverage the most due to secondary structures formed by longer microsatellites [38], [39], does not appear to have any significant effect on agreement between platforms, and the Illumina platform had no difficulties with poly-AC sequencing.

We have demonstrated that both short-read and long-read NGS platforms can be used to sequence short tandem repeats accurately, which makes it feasible to consider the use of these platforms in high-throughput genotyping. Nevertheless, the use of each platform is accompanied by a variety of challenges that should be considered when scoping such an experiment. First, it appears the major consideration for achieving high accuracy and rare microsatellite variant detection is sufficient read depth coverage. This might be a challenge because each platform generates a consistent pattern of non-uniform sequence coverage [37], which may affect some types of short tandem repeats more than others. For example, our study showed that, in 454-sequencing AC repeats were covered by significantly fewer reads than AT repeats. Second, achieving high read coverage per locus should be balanced by maximal throughput to provide significant time and cost advantage of these technologies. The last, but not the least, consideration is the choice of alignment techniques as these can impact the ability to identify microsatellite variations in various ways [6], [22].

## Supporting Information

Table S1 Summary of microsatellite variant data and statistics from Sanger, GS-FLX and Illumina platforms. *Indel variant* number refers to the number of repeat units deleted or inserted; negative number refers to a deletion; positive number refers to an insertion. *Var* refers to the observed number of reads for the particular variant, while *non-var* refers to the number of remaining reads covering the variant position; i.e. total coverage minus variant reads at the given position. *P used* refers to a standard chi-square test, except when (i) expected read counts were less than one and we used Monte-Carlo simulation to calculate P values or (ii) the observed number of reads minus the expected number was ≥ the expected number and we used a log-likelihood ratio goodness of fit test. P values for the Illumina platform are for three-way comparisons between Sanger, Illumina and 454-sequencing. Significance: * statistically significant values unadjusted for multiple tests (P<0.05), ** statistically significant values after Benjamini-Hochberg correction for multiple tests, *** statistically significant values after Bonferroni correction.
(XLSX)
